# Fossilization causes organisms to appear erroneously primitive by distorting evolutionary trees

**DOI:** 10.1038/srep02545

**Published:** 2013-08-29

**Authors:** Robert S. Sansom, Matthew A. Wills

**Affiliations:** 1 Faculty of Life Sciences, University of Manchester, Manchester M13 9PT, UK; 2 Department of Biology and Biochemistry, University of Bath, Bath, BA2 7AY, UK

## Abstract

Fossils are vital for calibrating rates of molecular and morphological change through geological time, and are the only direct source of data documenting macroevolutionary transitions. Many evolutionary studies therefore require the robust phylogenetic placement of extinct organisms. Here, we demonstrate that the inevitable bias of the fossil record to preserve just hard, skeletal morphology systemically distorts phylogeny. Removal of soft part characters from 78 modern vertebrate and invertebrate morphological datasets resulted in significant changes to phylogenetic signal; it caused individual taxa to drift from their original position, predominately downward toward the root of their respective trees. This last bias could systematically inflate evolutionary rates inferred from molecular data because first fossil occurrences will not be recognised as such. Stem-ward slippage, whereby fundamental taphonomic biases cause fossils to be interpreted as erroneously primitive, is therefore a ubiquitous problem for all biologists attempting to infer macroevolutionary rates or sequences.

Evolutionary biology aims to reveal the nature of macroevolutionary processes and therefore seeks answers to the following fundamental questions: Over what timescale does macroevolutionary change occur, and how do major clades originate and diverge? The first of these questions is usually addressed by calibrated rate studies, typically applying likelihood models to molecular data. Molecular clock analyses can draw on vast repositories of sequence data, but the only reliable way to apply an absolute timescale is to date and calibrate at least some divergences using the ages of fossils^1^,^2^,^3^,^4^. As to questions concerning major clade originations and transitions, fossils are the only source of *direct* evidence; they document historical sequences of character change and bridge morphological gaps within extinct branches of the tree of life^5^ (for example, how did fish evolve into tetrapods or dinosaurs evolve wings?). Modern taxa are distant from these transitions meaning that long evolutionary histories and large gaps exist between them and their ancient cladogeneses. Extinct taxa proximate to those events and enable reconstruction of morphological changes and transitions during these historic events. In order to address deep macroevolutionary questions, it therefore becomes vitally important to place fossils accurately within phylogenies. Without this, molecular clocks will be incorrectly calibrated, morphological sequences of change will be oversimplified, and our understanding of evolutionary processes will be flawed.

The phylogenetic affinities of fossils are almost universally inferred using morphological characters optimised with parsimony algorithms. Fossils, however, yield only a small and predictably restricted set of anatomical features, specifically those that survive the process of fossilization (usually shells, teeth and bones). The majority of extinct morphology is therefore lost to decay and decomposition and unavailable for study. It is currently not known if, or how, this systematic data loss and preservation bias affects our ability to reconstruct the phylogenetic relationships of most fossil taxa. Problematic fossilization biases have been experimentally demonstrated to distort our understanding of vertebrate origins; the decay of characters in entirely soft-bodied living chordates occurs in a systematic order, whereby the most derived synapomorphies diagnosing the crown are lost before more general, plesiomorphic characters^6^,^7^,^8^. Decay biases therefore cause early chordate and vertebrate fossil taxa to lack defining apomorphies. Unless fossilization processes (taphonomy) are taken into account, these fossils are artificially displaced to lower branches on the tree, towards, on even in, the stem of the crown clade to which they actually belong^9^. If fossils are misplaced in this way, it is likely that other candidate first occurrences for clades will be younger, thereby causing divergence dates to be underestimated; low fossil placement in a phylogeny therefore causes higher inferred rates of evolution (Fig. 1c). It remains unclear, however, whether this phenomenon of ‘stem-ward slippage' is restricted to chordates or is more widespread across other animal groups and thus whether it constitutes a serious and systemic problem for macroevolutionary studies.

The large amount of missing data afflicting fossils need not represent a problem in itself. The introduction of missing data entries into simulated data rarely obfuscates the phylogenetic placement of taxa in simulations^10^,^11^,^12^,^13^,^14^,^15^,^16^,^17^,^18^. The amount and quality of data that *are* available is usually much more important, although the situation can be more complex in some likelihood frameworks^19^,^20^. Missing data in the fossil record are, however, fundamentally non-random – it is both structured, and systematically distributed. Concentrating missing data entries in simulated taxa^14^ or characters^15^ can mimic some of the effects of fossilisation; simulations, however, oversimplify reality because their characters are effectively independent and they cannot take account of the markedly different preservation potentials of different tissue types.

For most animal groups, a simple distinction can be drawn between ‘hard', readily-fossilizable biomineralized structures (bones, teeth, shells) and ‘soft', less fossilizable non-biomineralized tissues (muscles, nerves, integument etc.). We use this distinction here to test the role of fossilization filters in shaping phylogenies and the evolutionary conclusions drawn from them. Firstly, we test the null hypothesis that simulated fossilization filters (i.e. the removal of soft part character data) cause change in inferred relationships no more than the removal of the same number of characters at random. The percentage of nodes recovered from the original, total evidence, strict consensus tree is used as a benchmark of original phylogenetic signal recovery. Secondly, we test the null hypothesis that individual extant taxa subjected to systematic fossilization filters have an equal probability of shifting ‘up' or ‘down' a phylogeny relative to the root. As such, we test whether stem-ward slippage is a ubiquitous phenomenon, and whether fossilization filters cause extinct taxa to move within a phylogeny in a consistent direction.

Fossil taxa have, by definition, already been subjected to character preservation biases. As such, it is difficult to test the above hypotheses using palaeontological data. Our solution is to apply hypothetical fossilization filters to data from extant clades. Using this approach, it is possible to compare the effects of simulated fossilization (systematic removal of soft non-biomineralized characters), with the effects of the random removal of the same number of characters. Tests were applied to 78 phylogenetic data matrices of disparate vertebrate and invertebrate clades, representing over 2000 taxa.

## Results

**Figure 1:**
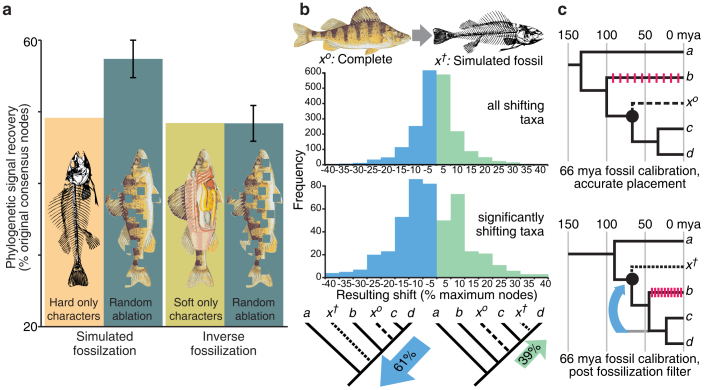
Results of simulated fossilization analyses. (a) Node recovery test, indicating significantly lower phylogenetic signal recovery for simulated fossilization searches (hard characters only) vs random missing characters in the same amount, but not for inverse-fossilization searches (soft characters only). Node recovery is the proportion of 2022 original strict consensus nodes recovered with systematic missing data or random missing data (the later being an average of 500 iterations with twice standard deviation error bars); (b) Taxon shift test, where soft characters are removed from individual taxa to simulate fossilization. Histograms represent shift from original position (*x ^o^*), to new position (*x*^†^) relative to the root for all simulated fossil taxa that moved (above) and simulated fossil taxa that exhibit significant shift, given random missing data (below). In the case of the later, 61% of 491 taxa shift significantly *down* phylogenies, from their original position toward the root, vs 39% which shift significantly *up*; (c) the effect of a downward shift of a fossil taxa in a phylogeny on estimates of rates of evolution and inferences of timing of evolutionary events (pink bars represent evolutionary changes e.g. DNA base pair change or acquisition of a morphological character). Images in Figure 1a adapted from Gilbert^34^ and Duane^35^.

Relatively few individual data matrices failed the node recovery test; 11 of the 78 datasets had P < 0.05, and thus significant loss of phylogenetic signal with the deletion of soft part characters. This was, however, significantly more (P = 0.002) than would be expected given the number of datasets tested (about 4). This indicates that the targeted deletion of soft-part characters (simulated fossilization) reduced the recovery of nodes more than the deletion of similar numbers of characters at random. When results from across data matrices were combined, the result was even more striking (Fig. 1a). Of the aggregate 2022 nodes in all of the original strict consensus trees, 49% were recovered when all taxa had soft parts removed whilst 57% were recovered when taxa had the same number of characters deleted at random. This difference was highly significant (not one of 500 random character deletion replicates had lower node recovery than the simulated fossilization aggregate, giving P = 0.002). Simulated fossilization filters were therefore significantly worse at recovering the relationships of the original total evidence consensus than the random deletion of the same numbers of characters. This contrasted markedly with the results of the inverse test, namely the preferential removal of hard characters. The same fraction of nodes (48%) was recovered when deleting hard part characters as when deleting the same number of characters at random (259 of 500 random character deletion replicates had lower average node recovery than the ‘inverse fossilization' aggregate, giving P = 0.52). This pattern of palaeontological bias is robust and not dependent on a few large data matrices; all of 50 jackknifed re-samplings (where each of the 78 data matrices was included with 50% probability) retained significant differences (P < 0.05) between the effects of simulated fossilization and random character deletion. When deleting hard-part characters (inverse fossilization), by contrast, 43 of 50 jackknifed re-samplings remained non-significant.

**Figure 2:**
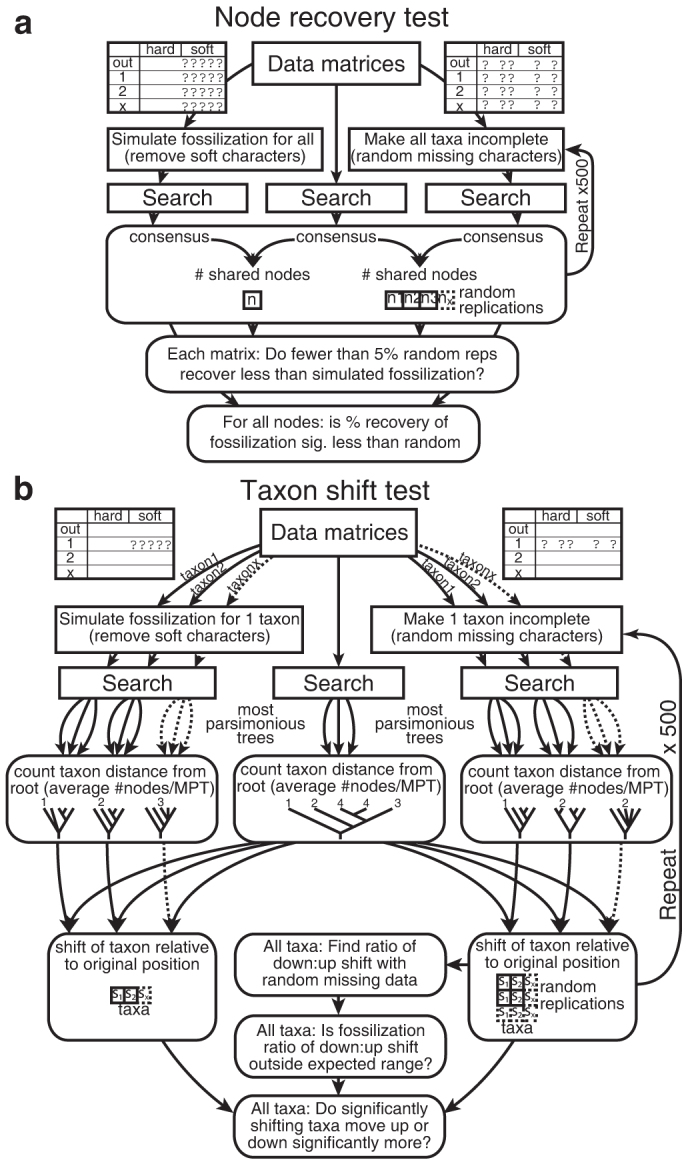
Schematic protocol of analyses. (a) Node recovery test; (b) Taxon shift test. Scripts for tests are available online (Supplementary 1–2).

Regarding the effect of missing data on individual taxa, the null expectation for a perturbed single terminal taxon is an equal probability of displacement up or down a phylogeny, toward or away from the root in a fully resolved tree (Fig. 1b, Fig. 2b). The taxon shift test confirmed this null for taxa perturbed by the introduction of random missing data. Of those taxa found to move relative to their original position, 49% moved down while 51% moved up (P = 0.75 for binomial test with *n* = 2124). By contrast, of the 2036 taxa that shifted position when soft characters were removed, 1071 moved down (53%), and 965 (47%) moved up. These frequencies differed significantly from those observed in random character deletion experiments (log likelihood ratio test; G = 5.367, P = 0.021) and also from the expected null (binomial test; P = 0.010). Of those 2036 simulated fossil taxa, 491 (24%) had a significant shift in position i.e. the magnitude of shift in position when soft characters were removed was seen in less than 5% of the 500 random missing data replicates. This is far more than the 5% we would expect by chance (binomial test, P = 4.2 × 10^−187^). Within those significantly shifting taxa, an even stronger bias towards downward movement was observed; of the 491 significantly shifting taxa, 61% moved down the tree, toward the root, whilst 39% moved up (Fig. 1b). This is highly significant (binomial test, P = 1.2 × 10^−6^ given a 0.5 null probability of downward movement). The same significant differences between simulated fossilization and random missing data and significant downward shift of taxa upon removal of soft characters was also observed in tests using traditional tree searches, or using patristic distances to root rather than numbers of nodes to root (see supplementary).

Simulated fossilization filters were therefore found to result in preferential and statistically significant shifts of taxa from their original position, predominately towards the basal node of their phylogeny. Our tests used these distances averaged for all most parsimonious, fully resolved trees, with no branch collapsing. As such, the preferential displacement of taxa toward the root cannot be related to the reduced resolution observed in the node recovery test, and is a distinct phenomenon.

## Discussion

Fossils are, by their very nature, incomplete. Whilst missing data in itself should not present a problem for reconstruction of phylogeny^13^,^14^,^15^,^16^,^17^,^18^,^21^, the results presented here demonstrate that the incompleteness particular to fossils – absence of soft-tissues – causes additional and systematic errors that could not be predicted from simulations alone. Not only does the preferential deletion of soft-part characters cause significantly more loss of the original phylogenetic relationships than the random deletion of characters, but it systematically shifts the reconstructed affinity of a fossil organism. When individual taxa are subjected to such simulated fossilization, they are significantly more likely to be displaced from their original position compared to random missing data. What is more, they are significantly more likely to be displaced towards the root of the tree than away from it. Fossils reconstructed as primitive, stem-group taxa may, therefore, actually have been derived members of the crown-group, spuriously displaced to the stem as an artefact of data loss during fossilization. This phenomenon of stem-ward slippage is much more than an inconvenience for those interested in the relationships of fossils from a palaeobiological perspective; it is the position of fossil taxa in trees relative to the root and extant taxa that is of central importance for studies inferring rates and sequences of macroevolutionary change. Specifically, fossils enable the accurate calibration of molecular clocks by defining minimum clade divergence times. Stem-ward slippage does not change the absolute age of a fossil, but it does change its inferred position in the tree. Any shift in fossil placement therefore has the potential to misplace calibration points within molecular trees (Fig. 1c) and makes it harder to identify the real first occurrence of a clade. Stemward slippage will result in lower calibration points and thus systematically distorts evolutionary rates, giving a narrower timeframe for evolutionary events to occur (Fig. 1c). Unless taphonomic factors are taken into account, it is likely that evolutionary rates of change, both molecular and morphological, will be overestimated (Fig. 1c). Similar problems will afflict studies attempting to determine the sequence of character change in stem lineages, and thus the nature of cladogenesis. Purported stem-group fossils may not reveal accurately the stages in the origin of clades, but be mere artefacts of fossilization biases and subsequent erroneous reconstruction.

The calibration problems demonstrated above are properly seen in the context of the errors inherent in determining first fossil occurrences more generally. All such dates are necessarily provisional and subject to revision: typically downwards as older fossil exemplars are discovered and documented. Some clock methods therefore specify likelihood functions around their point calibrations, admitting much greater potential for underestimated than overestimated ages. These methods are therefore already designed to take account of calibration point *underestimates*, such that the systematic overestimation of first occurrence ages (believing a group to originate earlier than it did) is much the more problematic type of error^1^. J B S Haldane famously noted that his belief in evolution would be shattered were someone to discover a rabbit in the Precambrian^22^. His comment reflects an intuitive distrust of radically revised *first* occurrence dates for derived groups (in contrast to an easy acceptance of the discovery of relict, living ‘fossil' *last* occurrences tens or hundreds of millions of years after their youngest fossil relatives). This, in turn, reflects the belief that the order of first fossil occurrences should be congruent with the order in which groups branch phylogenetically. Our results show that the removal of soft part character data makes the placement of taxa (either up or down the tree) significantly more labile than would be expected; 24% of the simulated fossil taxa that shift, shift significantly more than expected given random character loss. As such, fossilization filters causes appreciable displacement of taxa, irrespective of the direction of the movement. In this context, we note that significant *crownward* slippage (although less common than stemward slippage) occurred in 10% of all shifting simulated taxa. Such displacements could have the effect of making a stem representative appear to be part of a crown group, thereby potentially pulling the first occurrence date *downwards*; precisely the type of distortion with which relaxed clock methods are not optimally designed to contend.

Regarding the direction of displacement, the significant bias towards stemward slippage in simulated fossilization is relatively small (53% stemward versus 47% crownward), but the bias towards stemward slippage is much larger for those taxa that exhibit significant shift when compared to random incompleteness (61% versus 39%). The extent to which these displacements constitute a problem for clock and transition studies will ultimately depend upon the weight that is placed upon them in any given study. Where displaced fossils are utilised as one of just a small handful of calibration points, the greatest distortions are likely to ensue. We also note that the search for earliest exemplars can often result in the identification of fragmentary or poorly preserved material. Groups often originate at low diversity and with individuals of small size^23^ that will lack most of the diagnostic features of the crown clade. Earliest exemplars in real empirical data sets may therefore be less complete and more volatile than those in our simulations. An exception to this incompleteness is fossils from Konservat-Lagerstätten, but here different biases need to be taken into account. In fact, stem-ward slippage resulting from fossilization was first observed in exceptionally preserved early chordates where it results from decay biases within soft tissues^6^,^7^. Under these circumstances, systematic decay of anatomical features during fossilization can distort interpretation of the affinity of fossil taxa because taphonomic loss is easily conflated with phylogenetic absence. The results presented here indicate a more problematic effect over and above this; even where uncertainty is coded as such (? rather than 0), there can still be a residual tendency toward spurious migration of extinct taxa down phylogenetic trees. Hence, fossilization tends to degrade phylogenetic signal in precisely those characters that are most valuable for yielding an accurate, resolved tree. This observation was consistent across vertebrate and invertebrate taxa, and was based on matrices compiled at different taxonomic levels. Of the reptile datasets, for example, one is a family level analysis of all squamates^24^ and another an analysis of 93 species of the same genus of spiny lizard^25^. What is more, it seems that stem-ward slippage is a more ubiquitous phenomenon, afflicting the fossil record at a more fundamental level: all animals with biomineralized skeletons.

Hard and soft part characters evidently do not convey a homogeneous phylogentic signal. Marked and significant changes to inferred phylogenies only occur with the removal of soft-part characters (simulating the palaeontological case), and not with the removal of hard part characters (Fig. 1a). As such, synergy between the information provided by hard and soft characters is unlikely to be a factor behind the problem of palaeontological biases. One solution is to focus on morphological data from extant organisms and analyze them in the light of fossilization filters. Analysis of larger compilations of zoological data matrices will identify those characters and character types that contain most homoplasy, and crucially, highlight those subsets or modules of biomineralized characters that are most consistent with total evidence and molecular data^26^,^27^. It will then be possible to promote the use of such characters in palaeontological studies, enabling systematists to compare their results with and without controls for palaeontological biases. Until this point, we advocate caution when reconstructing and interpreting the phylogenetic relationships of fossil taxa, and a careful consideration of the impact of missing data when doing so.

## Methods

### Data collation and editing

Published morphological data matrices of diverse extant bilaterian groups containing a mixture of biomineralized and non-biomineralized morphological characters were compiled from the literature (Google, Google Scholar and Web of Knowledge searches for “*clade* phylogeny”, references therein, Cracraft and Donoghue^28^ and references therein). A straightforward binary distinction between biomineralized (hard) and non-biomineralized (soft) tissues was used to classify characters into those that are readily fossilizable versus those that are generally lost during fossilization. Only matrices containing a specified outgroup taxon were used.

Matrices were edited from their original published form by removing uninformative characters, extinct taxa and taxonomic equivalents^29^. Missing data were balanced between partitions by removing taxa with > 30% missing entries or characters with more than > 50% missing entries from matrices with a difference of 10% or more missing data between partitions. Further thresholds were set for the minimum number of characters (30), the minimum number of taxa (10), and the minimum and maximum ratios of hard:soft characters (0.20–0.80). These procedures resulted in 78 exclusively neontological data matrices, with balanced proportions of biomineralized and non-biomineralized characters containing similar amounts of missing data. As analyses of morphology (palaeontological and neontological) nearly always utilize parsimony methods for phylogenetic reconstruction, parsimony is applied here. Datasets were analyzed using TNT (Tree analysis using new technologies^30^).

### Node recovery test

The removal of any characters from a cladistic matrix has the potential to reduce the resolution of the strict consensus of the optimal trees derived from it. In the context of fossilization filters, we focused on the effect of removing less fossilizable, soft-part characters. The impact of simulated fossilization (deleting non-biomineralized characters, also termed pseudoextinction^31^,^32^,^33^) was compared with the effects of removing the same number of characters at random (protocol outlined in Fig. 2a).The original strict consensus tree for complete taxa (our benchmark) was found by subjecting all characters to a heuristic search (100 random additions and TBR branch swapping, holding 1000 trees per replication and a maximum of 10000 trees). This consensus was compared with the strict consensus resulting from searches (same settings) in which all non-biomineralized characters were removed (i.e. retaining only ‘hard' characters). The number of nodes common to the strict consensus of the soft characters only search and the original consensus, divided by the number of nodes in the benchmark consensus, provided an index of ‘node recovery' (i.e. the fraction of the original nodes recovered). The same comparison was made between the original, benchmark tree and consensus trees from 500 searches with random character deletion (deleting the same number of characters randomly). This yielded a distribution of node recovery indices from 500 random deletion exercises. The simulated fossilization node recovery value was deemed to be significantly different from this distribution if it lay in the 5% tail. For comparison, a precisely analogous ‘inverse fossilization' test was performed using deletion of biomineralized, hard characters (coupled with 500 random deletions of the same number of characters). All tests and metrics were implemented using TNT scripts written by RSS (Supplementary 1–2).

### Taxon shift test

The node recovery test treats all of the taxa in a data matrix as incomplete (either randomly or systematically) and therefore cannot provide information about the movement of taxa with missing data relative to other taxa. In order to investigate this phenomenon, it was necessary to apply simulated fossilization and random missing data to individual taxa, whilst leaving the rest of the matrix intact (protocol outlined in Fig. 2b). The original most parsimonious trees (MPTs) for the complete matrix were found using TNT searches with new technology (i.e. *xmult* at level 5, keeping all trees and multiplying by fusing, Supplementary 2) as well as traditional searches (same settings as node recovery test but with no branch collapsing, Supplementary 2). The original MPTs were then used to calculate the average distance of each taxon from the basal node (expressed as the average number of intervening nodes between the terminal and the root for all MPTs). Each taxon in turn was simulated as a fossil (all non-biomineralized characters for that taxon were replaced with “?”), a new search performed, and the resulting MPTs used to recalculate the new mean distance of that taxon from the basal node. For comparison, each taxon (in turn) was made randomly incomplete (the same number of characters deleted) 500 times, prior to parsimony searches that yielded distributions of mean distances from the basal node. For each taxon in each data matrix it was therefore possible to identify the distance (mean number of nodes or mean number of steps across all MPTs) and direction (up or down relative to the root) that a taxon was displaced from its original position when missing soft characters, and the distribution of mean distances and directions that a taxon moved from its original position when made randomly incomplete 500 times (Supplementary 3).

## Author Contributions

R.S.S. conceived the study, collected data and performed analyses. R.S.S. designed the analysis with contribution from M.A.W., R.S.S. and M.A.W. both wrote the manuscript.

## Supplementary Material

Supplementary Information Supplementary 1 and 2

Supplementary Information Supplementary 3
