# Supplementary material for: Fossilization causes organisms to appear erroneously primitive by distorting evolutionary trees
Source: Sci Rep. 2013 Aug 29;3:2545. doi: 10.1038/srep02545 (PMC3756334; doi:10.1038/srep02545)
Supplement: Supplementary Information — Supplementary 1 and 2 [file srep02545-s1.doc]

**SUPPLEMENTARY INFORMATION 1 and 2:**

**Fossilization causes organisms to appear erroneously primitive by distorting evolutionary trees**

Robert S. Sansom*^1,2^ & Matthew A. Wills^2^

^1^Faculty of Life Sciences, University of Manchester, Manchester M13 9PT, UK

^2^Department of Biology and Biochemistry, University of Bath, Bath, BA2 7AY, UK

/** SUPPLEMENTARY 1, TNT script for Node Recovery Test**/

macro-;

macro*3 300000;

macro[ 2000000;

macro=;

/** NodeRed500t Node Recovery Test, Robert Sansom**/

ccode [ .;

hold 10000;

log $dataset<.NRt.log;

tsave* $dataset<.NRt.tre;

collapse 3; collapse [;

/* normal search and consensus*/

mult=tbr replic 100 hold 1000;

if (ntrees == 0)

save/;

else

nelsen *;

save/;

end

keep 0;

/* hard only search and consensus*/

ccode ] {soft };

mult= tbr replic 100 hold 1000;

nelsen*;

save/;

keep 0;

tsave/;

collapse 0;

/* hard only node recovery */

proc $dataset<.NRt.tre;

tsave* $dataset<.NRt.tre;

nelsen * 0 1;

save . ;

tnodes;

ccode[.;

keep 0;

/* soft only search and consensus */

collapse 3; collapse [;

ccode ] .;

ccode [ {soft};

mult=tbr replic 100 hold 1000;

nelsen*;

save/;

keep 0;

tsave/;

/*soft only node recovery */

proc $dataset<.NRt.tre;

tsave* $dataset<.NRt.tre;

nelsen * 0 3;

save . ;

tnodes;

ccode [.;

tsave/;

collapse 0;

keep 0;

/* how many characters in group? */

var : nsoft nhard;

set nsoft 0; set nhard 0;

loop 0 nchar;

if (isinxgroup [ 0 #1 ])

set nsoft ++ ;

else

set nhard ++ ;

end

stop

/* Random removal of nsoft characters and construction of trees */

proc $dataset<.NRt.tre;

tsave* $dataset<.NRt.tre;

save.;

sil=console;

report-;

collapse 3; collapse [;

keep 0;

rseed *;

loop 0 499;

progress #1 500 Progress of 500 searches with nsoft random deletion;

rseed+1;

xgroup =1 (random) * 'nsoft';

ccode ]{random };

mult= tbr replic 100 hold 1000;

nelsen *;

save/;

ccode [ . ;

keep 0;

stop

tsave/;

collapse 0;

quote a;

/* Nodes recovery random trees */

proc $dataset<.NRt.tre;

tsave * $dataset<.NRt.tre;

save.;

loop 5 504

nelsen * 0 #1;

save/;

stop

tsave/;

report=;

sil-all;

quote b;

keep 0;

/* Random removal of nhard characters and construction of trees */

proc $dataset<.NRt.tre;

tsave* $dataset<.NRt.tre;

save.;

sil=all;

report-;

collapse 3; collapse [;

keep 0;

rseed *;

quote c;

loop 0 499;

progress #1 500 Progress of 500 searches with nhard random deletion;

rseed+1;

xgroup =1 (random) * 'nhard';

ccode ]{random };

mult= tbr replic 100 hold 1000;

nelsen *;

save/;

ccode [ . ;

keep 0;

stop

tsave/;

collapse 0;

/* Nodes recovery random trees */

proc $dataset<.NRt.tre;

tsave * $dataset<.NRt.tre;

save.;

loop 1005 1504

nelsen * 0 #1;

save/;

stop

report=;

sil-all;

/* Output results */

tsave/;

tnodes;

var : hardnodes softnodes;

set hardnodes tnodes [2];

set softnodes tnodes [4];

quote hardnodes 'hardnodes' softnodes 'softnodes';

var : nodessharedrh[500] nodessharedrs[500] temp;

set temp 505;

loop 0 499

set nodessharedrh [#1] tnodes ['temp'];

set temp++;

stop

var nodessharedrh*;

set temp 1505;

loop 0 499

set nodessharedrs [#1] tnodes ['temp'];

set temp++;

stop

var nodessharedrs*;

var : nodessharedrhavg;

set temp 0;

loop 0 499

set temp ('temp'+'nodessharedrh[#1]');

stop

set nodessharedrhavg ('temp'/500);

var : nodessharedrsavg;

set temp 0;

loop 0 499

set temp ('temp'+'nodessharedrs[#1]');

stop

set nodessharedrsavg ('temp'/500);

quote average shared nodes for nsoft characters missing 'nodessharedrhavg';

quote average shared nodes for nhard characters missing 'nodessharedrsavg';

var : randomshorth;

set randomshorth 0;

loop 0 499

if ('nodessharedrh [#1]' <= 'hardnodes')

set randomshorth ++;

end

stop

var : randomshorts;

set randomshorts 0;

loop 0 499

if ('nodessharedrs [#1]' <= 'softnodes')

set randomshorts ++;

end

stop

var : ph;

set ph (('randomshorth'+1)/ 501);

var : ps;

set ps (('randomshorts'+1)/ 501);

var: a b c d e;

set a tnodes[0]; set b tnodes[1]; set c tnodes[2]; set d tnodes[3]; set e tnodes[4];

macfloat 0;

quote Original strict consensus nodes, extinct nodes, extinct nodes shared with original, soft only nodes, soft only nodes shared with original;

quote 'a', 'b', 'c', 'd', 'e';

quote Random shared nodes with nsoft characters missing;

quote 'nodessharedrh[ 0 - 499 ]' ;

quote Random shared nodes with nhard characters missing;

quote 'nodessharedrs[ 0 - 499 ]' ;

macfloat 1;

quote Average number of nodes shared with original for random reps nsoft missing 'nodessharedrhavg';

quote Average number of nodes shared with original for random reps nhard missing 'nodessharedrsavg';

macfloat 3;

quote 'randomshorth' of 500 random reps have fewer nodes than hard only ('hardnodes' nodes);

quote Giving p value of 'ph';

quote 'randomshorts' of 500 random reps have fewer nodes than soft only ('softnodes' nodes);

quote Giving p value of 'ps';

quote output strict nodes, extinct shared nodes, rand avg, ps ph;

quote 'a' 'c' 'nodessharedrhavg' 'ph' 'ps';

/* calculate distance from root for each internal node for original consensus, not including root, or node immediate to root */

var : firstnode endnode nintnodes start;

set start 0;

set firstnode (ntax+2);

set endnode (tnodes[0]+ntax+1);

set nintnodes ('endnode'-'firstnode');

/* alternative way */

var : nodesdownX['nintnodes'];

set start 0;

loop=distnode 'firstnode' 'endnode'

if ((distnode[0 #1 0]-2) == 0)

continue

end

set nodesdownX ['start'] (distnode[0 #1 0]-2);

set start ++;

stop

/* make that relative to maximum node distance from root */

var : maxnodedist; set maxnodedist 0;

loop=nodemax 0 ('nintnodes' -1)

if ('nodesdownX[#nodemax]' > 'maxnodedist')

set maxnodedist 'nodesdownX[#nodemax]';

end

stop

var : output['nintnodes' 1003];

loop=noderel 0 ('nintnodes' -1)

set output[#noderel 0] ('nodesdownX[#noderel]'/'maxnodedist');

stop

/* find nodes in orginial tree (0) present in hard only tree (1) */

set start 0;

loop=nodeEh 'firstnode' 'endnode'

if ((distnode[0 #1 0]-2) == 0)

continue

end

if (eqgroup[0 #nodeEh 1] > 0 )

set output['start' 1] 1;

else

set output['start' 1] 0;

end

set start ++;

stop

/* find nodes in orginial tree (0) present in soft only tree (3) */

set start 0;

loop=nodeEs 'firstnode' 'endnode'

if ((distnode[0 #1 0]-2) == 0)

continue

end

if (eqgroup[0 #nodeEs 3] > 0 )

set output['start' 2] 1;

else

set output['start' 2] 0;

end

set start ++;

stop

/*find nodes in original tree (0) present in random reps of nsoft mssing characters*/

set start 0;

var : rep;

loop=nodeR 'firstnode' 'endnode'

if ((distnode[0 #1 0]-2) == 0)

continue

end

set rep 3;

loop=treecomp 5 504

if (eqgroup[0 #nodeR #treecomp] > 0 )

set output ['start' 'rep'] 1;

else

set output ['start' 'rep'] 0;

end

set rep ++;

stop

set start ++;

stop

/*find nodes in original tree (0) present in random reps of nhard mssing characters*/

set start 0;

var : rep;

loop=nodeR 'firstnode' 'endnode'

if ((distnode[0 #1 0]-2) == 0)

continue

end

set rep 503;

loop=treecomp 1505 2004

if (eqgroup[0 #nodeR #treecomp] > 0 )

set output ['start' 'rep'] 1;

else

set output ['start' 'rep'] 0;

end

set rep ++;

stop

set start ++;

stop

quote Results for hi vs lo expressed as;

quote column 1 node distance from root in original consensus (number nodes from root relative to maximum);

quote column 2 Recovered in hard only consensus tree (1 yes, 0 no);

quote column 3 Recovered in soft only consensus tree (1 yes, 0 no);

quote column 4-503 Recovered in random reps of nsoft missing characters (1 yes, 0 no);

quote column 504-1003 Recovered in random reps of nsoft missing characters (1 yes, 0 no);

var output*;

log/;

tsave/;

proc/;

/** SUPPLEMENTARY 2, TNT script for Taxon Shift Test **/

macro-;

macro* 4 500000;

macro [ 2000000;

macro=;

/** NRIND11, Taxon Shift Test, Robert Sansom (tree search with new technologies) **/

collapse 0;

hold 10000;

log $dataset<.NRIND11.log;

sect: slack 200;

xmult= level 5 keepall multiply;

unique;

report-;

sil=all;

macfloat 3;

/** calculate average distance from root for each taxon for original MPTs (not including taxon 0)**/

var : nodesdownavgO[(ntax+1)] nodesdownthis nodesdownall x y blength[(2*ntax+1)] lengthdownall lengthdownavgO[(ntax+1)];

set x (ntax+1); set y (ntrees+1);

loop=taxonO 0 ntax

progress #1 ntax Progress of node and length counting for 'y' trees;

set nodesdownall 0;

set lengthdownall 0;

loop=MPTO 0 ntrees

maketable + blength;

blength #MPTO;

maketable-;

travtree below #MPTO #taxonO nodesdownthis

set nodesdownall ++;

set lengthdownall ('lengthdownall'+'blength['nodesdownthis']');

endtrav

stop

set nodesdownavgO[#taxonO] ('nodesdownall'/(ntrees+1));

set lengthdownavgO[#taxonO] ('lengthdownall'/(ntrees+1));

stop

progress/;

keep 0;

/** create trees for hard only for each taxon **/

var : nodesdownavgE[(ntax+1)] nodesrelativeavgOE[(ntax+1)] lengthdownavgE[(ntax+1)] lengthrelativeavgOE[(ntax+1)];

loop=taxonE 1 ntax

progress #1 ntax Progress of hard only searches for each taxon ('y' trees);

proc $dataset;

loop=extinct 0 nchar;

if ( isinxgroup [0 #extinct])

xread=!#extinct #taxonE ?;

end

stop

xread!;

hold 10000;

xmult= level 5 keepall multiply;

unique;

set y (ntrees+1);

set nodesdownall 0;

set lengthdownall 0;

/**calculate average distance from root for #taxon hard only MPTs**/

loop=MPTE 0 ntrees

maketable + blength;

blength #MPTE;

maketable-;

travtree below #MPTE #taxonE nodesdownthis

set nodesdownall ++;

set lengthdownall ('lengthdownall'+'blength['nodesdownthis']');

endtrav

stop

set nodesdownavgE[#taxonE] ('nodesdownall'/(ntrees+1));

set nodesrelativeavgOE[#taxonE] ('nodesdownavgE[#taxonE]'-'nodesdownavgO[#taxonE]');

set lengthdownavgE[#taxonE] ('lengthdownall'/(ntrees+1));

set lengthrelativeavgOE[#taxonE] ('lengthdownavgE[#taxonE]'-'lengthdownavgO[#taxonE]');

keep 0;

stop

keep 0;

progress/;

proc $dataset;

var : i;

/***** How many characters in soft group? *****/

var : nsoft; set nsoft 0;

loop 0 nchar;

if (isinxgroup [ 0 #1 ])

set nsoft ++ ;

end

stop

/** create trees for random deletions for each taxon in turn**/

var : nodesdownavgR[(ntax+1) 500] nodesrelativeavgOR[(ntax+1) 500] lengthdownavgR[(ntax+1) 500] lengthrelativeavgOR[(ntax+1) 500];

keep0;

loop=taxonR 1 ntax

rseed*;

loop=randomrep 0 499

progress #2 500 Progress of random MD for taxon #1 of 'x' ('y' trees);

proc $dataset; rseed+1;

/** make random missing data and search **/

xgroup = 2 (random) * 'nsoft';

loop=psextinct 0 nchar;

if ( isinxgroup [2 #psextinct])

xread=!#psextinct #taxonR ?;

end

stop

xread!;

quote NUMBER OF TREE 'y';

hold 10000;

xmult= level 5 keepall multiply;

unique;

set y (ntrees+1);

/** count nodesdown to root for MPTs**/

set nodesdownall 0;

set lengthdownall 0;

loop=treeR 0 ntrees

maketable + blength;

blength #treeR;

maketable-;

travtree below #treeR #taxonR nodesdownthis

set nodesdownall ++;

set lengthdownall ('lengthdownall'+'blength['nodesdownthis']');

endtrav

stop

set nodesdownavgR[#taxonR #randomrep] ('nodesdownall'/(ntrees+1));

set nodesrelativeavgOR[#taxonR #randomrep] ('nodesdownavgR[#taxonR #randomrep]'-'nodesdownavgO[#taxonR]');

set lengthdownavgR[#taxonR #randomrep] ('lengthdownall'/(ntrees+1));

set lengthrelativeavgOR[#taxonR #randomrep] (('lengthdownall'/(ntrees+1)) - 'lengthdownavgO[#taxonR]');

stop

progress/;

stop

proc $dataset;

sil-all;

quote a;

/**maximum distance from root to exclude taxa on extreme tips (down only)**/

var : maxdistanceO y exclusions[ntax];

set maxdistanceO 0; set y 1; set exclusions [0] 0;

loop=taxC 1 ntax

if ('nodesdownavgO[#taxC]' > 'maxdistanceO')

set maxdistanceO 'nodesdownavgO[#taxC]';

end

stop

quote b;

/**then count up or down and whether moves, with exclusions for base and 2 tips**/

var : taxranddownup[(ntax+1) 2] thisdown thisup alldownOR allupOR allnullOR alldownOE allupOE allnullOE;

set alldownOR 0; set allupOR 0; set allnullOR 0; set alldownOE 0; set allupOE 0; set allnullOE 0;

loop=taxB 1 ntax

if ('nodesdownavgO[#taxB]' == 'maxdistanceO')

set taxranddownup[#1 0] 0;

set taxranddownup[#1 1] 0;

set exclusions['y'] #1;

set y++;

continue

end

if ('nodesdownavgO[#taxB]' == 3 )

set taxranddownup[#1 0] 0;

set taxranddownup[#1 1] 0;

set exclusions['y'] #1;

set y++;

continue

end

set thisdown 0; set thisup 0;

loop=repB 0 499

if ('nodesrelativeavgOR[#taxB #repB]' < 0)

set thisdown ++;

set alldownOR ++;

end

if ('nodesrelativeavgOR[#taxB #repB]' > 0)

set thisup ++;

set allupOR ++;

end

if ('nodesrelativeavgOR[#taxB #repB]' == 0)

set allnullOR ++;

end

stop

if ('nodesrelativeavgOE[#taxB]' < 0)

set alldownOE ++;

end

if ('nodesrelativeavgOE[#taxB]' > 0)

set allupOE ++;

end

if ('nodesrelativeavgOE[#taxB]' == 0)

set allnullOE ++;

end

set taxranddownup[#1 0] 'thisdown';

set taxranddownup[#1 1] 'thisup';

stop

quote c;

/**then count up or down and whether moves, without exclusions **/

var : taxranddownupx[(ntax+1) 2] alldownORx allupORx allnullORx alldownOEx allupOEx allnullOEx;

set alldownORx 0; set allupORx 0; set allnullORx 0; set alldownOEx 0; set allupOEx 0; set allnullOEx 0;

loop=taxC 1 ntax

set thisdown 0; set thisup 0;

loop=repC 0 499

if ('nodesrelativeavgOR[#taxC #repC]' < 0)

set thisdown ++;

set alldownORx ++;

end

if ('nodesrelativeavgOR[#taxC #repC]' > 0)

set thisup ++;

set allupORx ++;

end

if ('nodesrelativeavgOR[#taxC #repC]' == 0)

set allnullORx ++;

end

stop

if ('nodesrelativeavgOE[#taxC]' < 0)

set alldownOEx ++;

end

if ('nodesrelativeavgOE[#taxC]' > 0)

set allupOEx ++;

end

if ('nodesrelativeavgOE[#taxC]' == 0)

set allnullOEx ++;

end

set taxranddownupx[#taxC 0] 'thisdown';

set taxranddownupx[#taxC 1] 'thisup';

stop

quote d;

/** is position of extinct taxon (length) more or less than average of random missing data replicates?**/

var : thislengthall lengthdownavgavgR[(ntax+1)] taxlengthOElessavgOR taxlengthOEmoreavgOR taxlengthOEisavgOR;

set taxlengthOElessavgOR 0; set taxlengthOEmoreavgOR 0; set taxlengthOEisavgOR 0;

loop=taxC 1 ntax

set thislengthall 0;

loop=repc 0 499

set thislengthall ('thislengthall'+'lengthdownavgR[#taxC #repc]');

stop

set lengthdownavgavgR[#1] ('thislengthall'/500);

if ('lengthdownavgE[#1]' < ('thislengthall'/500))

set taxlengthOElessavgOR ++;

end

if ('lengthdownavgE[#1]' > ('thislengthall'/500))

set taxlengthOEmoreavgOR ++;

end

if ('lengthdownavgE[#1]' == ('thislengthall'/500))

set taxlengthOEisavgOR ++;

end

stop

quote e;

/** calculate if extinct taxa movement (nodes) is outside range of random missing data replicates, excluding base and tips **/

var : nodesrelativeout[(ntax+1)] n taxmovenonsig taxdownsig taxupsig;

set taxmovenonsig 0; set taxdownsig 0; set taxupsig 0;

loop=tax 1 ntax

if ('nodesrelativeavgOE[#tax]' == 0)

set nodesrelativeout[#tax] 2;

continue

end

if ('nodesdownavgO[#tax]' == 'maxdistanceO')

set nodesrelativeout[#tax] 2;

continue

end

if ('nodesdownavgO[#tax]' == 3 )

set nodesrelativeout[#tax] 2;

continue

end

set n 1;

if ('nodesrelativeavgOE[#tax]'< 0)

loop=rep 0 499

if('nodesrelativeavgOR[#tax #rep]' < 'nodesrelativeavgOE[#tax]')

set n++;

end

stop

set nodesrelativeout[#tax] ('n'/501);

if('n'<25)

set taxdownsig ++;

else

set taxmovenonsig ++;

end

end

if ('nodesrelativeavgOE[#tax]'> 0)

loop=rep 0 499

if('nodesrelativeavgOR[#tax #rep]' > 'nodesrelativeavgOE[#tax]')

set n++;

end

stop

set nodesrelativeout[#tax] ('n'/501);

if('n'<25)

set taxupsig ++;

else

set taxmovenonsig ++;

end

end

stop

quote f;

/** calculate if extinct taxa movement (nodes) is outside range of random missing data replicates, without exclusions **/

var : nodesrelativeoutx[(ntax+1)] taxmovenonsigx taxdownsigx taxupsigx;

set taxmovenonsigx 0; set taxdownsigx 0; set taxupsigx 0;

quote fz;

loop=taxx 1 ntax

quote fa#1;

set n 1;

if ('nodesrelativeavgOE[#taxx]' == 0)

set nodesrelativeoutx[#taxx] 2;

continue

quote fb#1;

end

if ('nodesrelativeavgOE[#taxx]'< 0)

loop=repx 0 499

if('nodesrelativeavgOR[#taxx #repx]' < 'nodesrelativeavgOE[#taxx]')

set n++;

end

stop

quote fc#1;

set nodesrelativeoutx[#taxx] ('n'/501);

if('n'<25)

set taxdownsigx ++;

else

set taxmovenonsigx ++;

end

quote fd#1;

end

if ('nodesrelativeavgOE[#taxx]'> 0)

loop=repx 0 499

if('nodesrelativeavgOR[#taxx #repx]' > 'nodesrelativeavgOE[#taxx]')

set n++;

end

stop

set nodesrelativeoutx[#taxx] ('n'/501);

if('n'<25)

set taxupsigx ++;

else

set taxmovenonsigx ++;

end

end

stop

quote g;

/** calculate if extinct taxa movement (length) is outside range of random missing data replicates **/

var : lengthrelativeout[(ntax+1)] taxmovenonsigl taxdownsigl taxupsigl;

set taxmovenonsigl 0; set taxdownsigl 0; set taxupsigl 0;

loop=taxl 1 ntax

set n 1;

if ('lengthrelativeavgOE[#taxl]' == 0)

set lengthrelativeout[#taxl] 2;

continue

end

if ('lengthrelativeavgOE[#taxl]'< 0)

loop=repl 0 499

if('lengthrelativeavgOR[#taxl #repl]' < 'lengthrelativeavgOE[#taxl]')

set n++;

end

stop

set lengthrelativeout[#taxl] ('n'/501);

if('n'<25)

set taxdownsigl ++;

else

set taxmovenonsigl ++;

end

end

if ('lengthrelativeavgOE[#taxl]'> 0)

loop=repl 0 499

if('lengthrelativeavgOR[#taxl #repl]' > 'lengthrelativeavgOE[#taxl]')

set n++;

end

stop

set lengthrelativeout[#taxl] ('n'/501);

if('n'<25)

set taxupsigl ++;

else

set taxmovenonsigl ++;

end

end

stop

quote h;

keep 0;

var : entsoft entsoftmiss enthard enthardmiss this;

set entsoft 0; set entsoftmiss 0; set enthard 0; set enthardmiss 0;

randtrees 1;

loop 0 ntax

loop 0 nchar

set this states [#2 #1 0];

if (isinxgroup [0 #2])

set entsoft ++;

if ('this' > 250)

set entsoftmiss ++;

end

else

set enthard++;

if ('this' > 250)

set enthardmiss ++;

end

end

stop

stop

quote i;

sil-all;

macfloat 2;

quote Average distance from root for each taxon in original MPTs (maximum is 'maxdistanceO');

var nodesdownavgO*;

quote Average number of nodes a taxon moves relative to original position when extinct;

var nodesrelativeavgOE*;

quote Average number of nodes a taxon moves relative to original position for random missing data replications;

var nodesrelativeavgOR*;

quote Average length from root for each taxon in original MPTs;

var lengthdownavgO*;

quote Average length a taxon moves relative to original position when extinct;

var lengthrelativeavgOE*;

quote Average of average lengths of taxa for random replications;

var lengthdownavgavgR*;

quote Average length a taxon moves relative to original position for random missing data replications;

var lengthrelativeavgOR*;

quote Taxa excluded from some counts, including max distance 'maxdistanceO';

var exclusions*;

macfloat 0;

quote Counts for each taxon of movement for missing data in 500 random reps, down and up with exclusions;

var taxranddownup*;

quote Counts for each taxon of movement for missing data in 500 random reps, down and up without exclusions;

var taxranddownupx*;

macfloat 4;

quote Significance values for movement of each taxon (2 for no movement);

var nodesrelativeout*;

macfloat 0;

quote -;

quote Output for 500 random missing data replications with exclusions node movement;

quote 'allnullOR' 'alldownOR' 'allupOR' a taxon moves no where, down or up;

quote Output for 500 random missing data replications without exclusions node movement;

quote 'allnullORx' 'alldownORx' 'allupORx' a taxon moves no-where, down or up;

quote Output for systematic extinction, node movement with exclusions;

quote 'allnullOE' 'alldownOE' 'allupOE' a taxon moves no-where, down or up for systematic hard only;

quote Output for systematic extinction, node movement without exclusions;

quote 'allnullOEx' 'alldownOEx' 'allupOEx' a taxon moves no-where, moves down, or up;

quote Significantly moving taxa (nodes) with exclusions;

quote 'taxmovenonsig' 'taxdownsig' 'taxupsig' non-significant, move down, move up;

quote Significantly moving taxa (nodes) without exclusions;

quote 'taxmovenonsigx' 'taxdownsigx' 'taxupsigx';

quote Change in length of extinct relative to average of random missing data reps;

quote 'taxlengthOEisavgOR' 'taxlengthOElessavgOR' 'taxlengthOEmoreavgOR' a taxon has no difference, is less (down) or more (up);

quote Significantly shifting taxa (length);

quote 'taxmovenonsigl' 'taxdownsigl' 'taxupsigl' non-significant movement, significant down, significant up;

quote 'entsoftmiss' entries of 'entsoft' soft entries are missing;

quote 'enthardmiss' entries of 'enthard' hard entries are missing;

report=;

log/;

proc/;

macro-;

macro* 4 500000;

macro [ 2000000;

macro=;

/** NRIND10, Taxon Shift Test, Robert Sansom (traditional search) **/

collapse 0;

hold 10000;

log $dataset<.NRIND10.log;

mult= tbr replic 100 hold 1000;

unique;

report-;

sil=all;

macfloat 3;

/** calculate average distance from root for each taxon for original MPTs (not including taxon 0)**/

var : nodesdownavgO[(ntax+1)] nodesdownthis nodesdownall x y blength[(2*ntax+1)] lengthdownall lengthdownavgO[(ntax+1)];

set x (ntax+1); set y (ntrees+1);

loop=taxonO 0 ntax

progress #1 ntax Progress of node and length counting for 'y' trees;

set nodesdownall 0;

set lengthdownall 0;

loop=MPTO 0 ntrees

maketable + blength;

blength #MPTO;

maketable-;

travtree below #MPTO #taxonO nodesdownthis

set nodesdownall ++;

set lengthdownall ('lengthdownall'+'blength['nodesdownthis']');

endtrav

stop

set nodesdownavgO[#taxonO] ('nodesdownall'/(ntrees+1));

set lengthdownavgO[#taxonO] ('lengthdownall'/(ntrees+1));

stop

progress/;

keep 0;

/** create trees for hard only for each taxon **/

var : nodesdownavgE[(ntax+1)] nodesrelativeavgOE[(ntax+1)] lengthdownavgE[(ntax+1)] lengthrelativeavgOE[(ntax+1)];

loop=taxonE 1 ntax

progress #1 ntax Progress of hard only searches for each taxon ('y' trees);

proc $dataset;

loop=extinct 0 nchar;

if ( isinxgroup [0 #extinct])

xread=!#extinct #taxonE ?;

end

stop

xread!;

hold 10000;

mult= tbr replic 100 hold 1000;

unique;

set y (ntrees+1);

set nodesdownall 0;

set lengthdownall 0;

/**calculate average distance from root for #taxon hard only MPTs**/

loop=MPTE 0 ntrees

maketable + blength;

blength #MPTE;

maketable-;

travtree below #MPTE #taxonE nodesdownthis

set nodesdownall ++;

set lengthdownall ('lengthdownall'+'blength['nodesdownthis']');

endtrav

stop

set nodesdownavgE[#taxonE] ('nodesdownall'/(ntrees+1));

set nodesrelativeavgOE[#taxonE] ('nodesdownavgE[#taxonE]'-'nodesdownavgO[#taxonE]');

set lengthdownavgE[#taxonE] ('lengthdownall'/(ntrees+1));

set lengthrelativeavgOE[#taxonE] ('lengthdownavgE[#taxonE]'-'lengthdownavgO[#taxonE]');

stop

keep 0;

progress/;

proc $dataset;

var : i;

/***** How many characters in soft group? *****/

var : nsoft; set nsoft 0;

loop 0 nchar;

if (isinxgroup [ 0 #1 ])

set nsoft ++ ;

end

stop

/** create trees for random deletions for each taxon in turn**/

var : nodesdownavgR[(ntax+1) 500] nodesrelativeavgOR[(ntax+1) 500] lengthdownavgR[(ntax+1) 500] lengthrelativeavgOR[(ntax+1) 500];

keep0;

loop=taxonR 1 ntax

rseed*;

loop=randomrep 0 499

progress #2 500 Progress of random MD for taxon #1 of 'x' ('y' trees);

proc $dataset; rseed+1;

/** make random missing data and search **/

xgroup = 2 (random) * 'nsoft';

loop=psextinct 0 nchar;

if ( isinxgroup [2 #psextinct])

xread=!#psextinct #taxonR ?;

end

stop

xread!;

hold 10000;

mult= tbr replic 100 hold 1000;

unique;

set y (ntrees + 1);

/** count nodesdown to root for MPTs**/

set nodesdownall 0;

set lengthdownall 0;

loop=treeR 0 ntrees

maketable + blength;

blength #treeR;

maketable-;

travtree below #treeR #taxonR nodesdownthis

set nodesdownall ++;

set lengthdownall ('lengthdownall'+'blength['nodesdownthis']');

endtrav

stop

set nodesdownavgR[#taxonR #randomrep] ('nodesdownall'/(ntrees+1));

set nodesrelativeavgOR[#taxonR #randomrep] ('nodesdownavgR[#taxonR #randomrep]'-'nodesdownavgO[#taxonR]');

set lengthdownavgR[#taxonR #randomrep] ('lengthdownall'/(ntrees+1));

set lengthrelativeavgOR[#taxonR #randomrep] (('lengthdownall'/(ntrees+1)) - 'lengthdownavgO[#taxonR]');

stop

progress/;

stop

proc $dataset;

sil-all;

quote a;

/**maximum distance from root to exclude taxa on extreme tips (down only)**/

var : maxdistanceO y exclusions[ntax];

set maxdistanceO 0; set y 1; set exclusions [0] 0;

loop=taxC 1 ntax

if ('nodesdownavgO[#taxC]' > 'maxdistanceO')

set maxdistanceO 'nodesdownavgO[#taxC]';

end

stop

quote b;

/**then count up or down and whether moves, with exclusions for base and 2 tips**/

var : taxranddownup[(ntax+1) 2] thisdown thisup alldownOR allupOR allnullOR alldownOE allupOE allnullOE;

set alldownOR 0; set allupOR 0; set allnullOR 0; set alldownOE 0; set allupOE 0; set allnullOE 0;

loop=taxB 1 ntax

if ('nodesdownavgO[#taxB]' == 'maxdistanceO')

set taxranddownup[#1 0] 0;

set taxranddownup[#1 1] 0;

set exclusions['y'] #1;

set y++;

continue

end

if ('nodesdownavgO[#taxB]' == 3 )

set taxranddownup[#1 0] 0;

set taxranddownup[#1 1] 0;

set exclusions['y'] #1;

set y++;

continue

end

set thisdown 0; set thisup 0;

loop=repB 0 499

if ('nodesrelativeavgOR[#taxB #repB]' < 0)

set thisdown ++;

set alldownOR ++;

end

if ('nodesrelativeavgOR[#taxB #repB]' > 0)

set thisup ++;

set allupOR ++;

end

if ('nodesrelativeavgOR[#taxB #repB]' == 0)

set allnullOR ++;

end

stop

if ('nodesrelativeavgOE[#taxB]' < 0)

set alldownOE ++;

end

if ('nodesrelativeavgOE[#taxB]' > 0)

set allupOE ++;

end

if ('nodesrelativeavgOE[#taxB]' == 0)

set allnullOE ++;

end

set taxranddownup[#1 0] 'thisdown';

set taxranddownup[#1 1] 'thisup';

stop

quote c;

/**then count up or down and whether moves, without exclusions **/

var : taxranddownupx[(ntax+1) 2] alldownORx allupORx allnullORx alldownOEx allupOEx allnullOEx;

set alldownORx 0; set allupORx 0; set allnullORx 0; set alldownOEx 0; set allupOEx 0; set allnullOEx 0;

loop=taxC 1 ntax

set thisdown 0; set thisup 0;

loop=repC 0 499

if ('nodesrelativeavgOR[#taxC #repC]' < 0)

set thisdown ++;

set alldownORx ++;

end

if ('nodesrelativeavgOR[#taxC #repC]' > 0)

set thisup ++;

set allupORx ++;

end

if ('nodesrelativeavgOR[#taxC #repC]' == 0)

set allnullORx ++;

end

stop

if ('nodesrelativeavgOE[#taxC]' < 0)

set alldownOEx ++;

end

if ('nodesrelativeavgOE[#taxC]' > 0)

set allupOEx ++;

end

if ('nodesrelativeavgOE[#taxC]' == 0)

set allnullOEx ++;

end

set taxranddownupx[#taxC 0] 'thisdown';

set taxranddownupx[#taxC 1] 'thisup';

stop

quote d;

/** is position of extinct taxon (length) more or less than average of random missing data replicates?**/

var : thislengthall lengthdownavgavgR[(ntax+1)] taxlengthOElessavgOR taxlengthOEmoreavgOR taxlengthOEisavgOR;

set taxlengthOElessavgOR 0; set taxlengthOEmoreavgOR 0; set taxlengthOEisavgOR 0;

loop=taxC 1 ntax

set thislengthall 0;

loop=repc 0 499

set thislengthall ('thislengthall'+'lengthdownavgR[#taxC #repc]');

stop

set lengthdownavgavgR[#1] ('thislengthall'/500);

if ('lengthdownavgE[#1]' < ('thislengthall'/500))

set taxlengthOElessavgOR ++;

end

if ('lengthdownavgE[#1]' > ('thislengthall'/500))

set taxlengthOEmoreavgOR ++;

end

if ('lengthdownavgE[#1]' == ('thislengthall'/500))

set taxlengthOEisavgOR ++;

end

stop

quote e;

/** calculate if extinct taxa movement (nodes) is outside range of random missing data replicates, excluding base and tips **/

var : nodesrelativeout[(ntax+1)] n taxmovenonsig taxdownsig taxupsig;

set taxmovenonsig 0; set taxdownsig 0; set taxupsig 0;

loop=tax 1 ntax

if ('nodesrelativeavgOE[#tax]' == 0)

set nodesrelativeout[#tax] 2;

continue

end

if ('nodesdownavgO[#tax]' == 'maxdistanceO')

set nodesrelativeout[#tax] 2;

continue

end

if ('nodesdownavgO[#tax]' == 3 )

set nodesrelativeout[#tax] 2;

continue

end

set n 1;

if ('nodesrelativeavgOE[#tax]'< 0)

loop=rep 0 499

if('nodesrelativeavgOR[#tax #rep]' < 'nodesrelativeavgOE[#tax]')

set n++;

end

stop

set nodesrelativeout[#tax] ('n'/501);

if('n'<25)

set taxdownsig ++;

else

set taxmovenonsig ++;

end

end

if ('nodesrelativeavgOE[#tax]'> 0)

loop=rep 0 499

if('nodesrelativeavgOR[#tax #rep]' > 'nodesrelativeavgOE[#tax]')

set n++;

end

stop

set nodesrelativeout[#tax] ('n'/501);

if('n'<25)

set taxupsig ++;

else

set taxmovenonsig ++;

end

end

stop

quote f;

/** calculate if extinct taxa movement (nodes) is outside range of random missing data replicates, without exclusions **/

var : nodesrelativeoutx[(ntax+1)] taxmovenonsigx taxdownsigx taxupsigx;

set taxmovenonsigx 0; set taxdownsigx 0; set taxupsigx 0;

loop=taxx 1 ntax

quote fa#1;

set n 1;

if ('nodesrelativeavgOE[#taxx]' == 0)

set nodesrelativeoutx[#taxx] 2;

continue

quote fb#1;

end

if ('nodesrelativeavgOE[#taxx]'< 0)

loop=repx 0 499

if('nodesrelativeavgOR[#taxx #repx]' < 'nodesrelativeavgOE[#taxx]')

set n++;

end

stop

quote fc#1;

set nodesrelativeoutx[#taxx] ('n'/501);

if('n'<25)

set taxdownsigx ++;

else

set taxmovenonsigx ++;

end

quote fd#1;

end

if ('nodesrelativeavgOE[#taxx]'> 0)

loop=repx 0 499

if('nodesrelativeavgOR[#taxx #repx]' > 'nodesrelativeavgOE[#taxx]')

set n++;

end

stop

set nodesrelativeoutx[#taxx] ('n'/501);

if('n'<25)

set taxupsigx ++;

else

set taxmovenonsigx ++;

end

end

stop

quote g;

/** calculate if extinct taxa movement (length) is outside range of random missing data replicates **/

var : lengthrelativeout[(ntax+1)] taxmovenonsigl taxdownsigl taxupsigl;

set taxmovenonsigl 0; set taxdownsigl 0; set taxupsigl 0;

loop=taxl 1 ntax

set n 1;

if ('lengthrelativeavgOE[#taxl]' == 0)

set lengthrelativeout[#taxl] 2;

continue

end

if ('lengthrelativeavgOE[#taxl]'< 0)

loop=repl 0 499

if('lengthrelativeavgOR[#taxl #repl]' < 'lengthrelativeavgOE[#taxl]')

set n++;

end

stop

set lengthrelativeout[#taxl] ('n'/501);

if('n'<25)

set taxdownsigl ++;

else

set taxmovenonsigl ++;

end

end

if ('lengthrelativeavgOE[#taxl]'> 0)

loop=repl 0 499

if('lengthrelativeavgOR[#taxl #repl]' > 'lengthrelativeavgOE[#taxl]')

set n++;

end

stop

set lengthrelativeout[#taxl] ('n'/501);

if('n'<25)

set taxupsigl ++;

else

set taxmovenonsigl ++;

end

end

stop

quote h;

sil-all;

macfloat 2;

quote Average distance from root for each taxon in original MPTs (maximum is 'maxdistanceO');

var nodesdownavgO*;

quote Average number of nodes a taxon moves relative to original position when extinct;

var nodesrelativeavgOE*;

quote Average number of nodes a taxon moves relative to original position for random missing data replications;

var nodesrelativeavgOR*;

quote Average length from root for each taxon in original MPTs;

var lengthdownavgO*;

quote Average length a taxon moves relative to original position when extinct;

var lengthrelativeavgOE*;

quote Average of average lengths of taxa for random replications;

var lengthdownavgavgR*;

quote Average length a taxon moves relative to original position for random missing data replications;

var lengthrelativeavgOR*;

quote Taxa excluded from some counts, including max distance 'maxdistanceO';

var exclusions*;

macfloat 0;

quote Counts for each taxon of movement for missing data in 500 random reps, down and up with exclusions;

var taxranddownup*;

quote Counts for each taxon of movement for missing data in 500 random reps, down and up without exclusions;

var taxranddownupx*;

macfloat 4;

quote Significance values for movement of each taxon (2 for no movement);

var nodesrelativeout*;

macfloat 0;

quote -;

quote Output for 500 random missing data replications with exclusions node movement;

quote 'allnullOR' 'alldownOR' 'allupOR' a taxon moves no where, down or up;

quote Output for 500 random missing data replications without exclusions node movement;

quote 'allnullORx' 'alldownORx' 'allupORx' a taxon moves no-where, down or up;

quote Output for systematic extinction, node movement with exclusions;

quote 'allnullOE' 'alldownOE' 'allupOE' a taxon moves no-where, down or up for systematic hard only;

quote Output for systematic extinction, node movement without exclusions;

quote 'allnullOEx' 'alldownOEx' 'allupOEx' a taxon moves no-where, moves down, or up;

quote Significantly moving taxa (nodes) with exclusions;

quote 'taxmovenonsig' 'taxdownsig' 'taxupsig' non-significant, move down, move up;

quote Significantly moving taxa (nodes) without exclusions;

quote 'taxmovenonsigx' 'taxdownsigx' 'taxupsigx';

quote Change in length of extinct relative to average of random missing data reps;

quote 'taxlengthOEisavgOR' 'taxlengthOElessavgOR' 'taxlengthOEmoreavgOR' a taxon has no difference, is less (down) or more (up);

quote Significantly shifting taxa (length);

quote 'taxmovenonsigl' 'taxdownsigl' 'taxupsigl' non-significant movement, significant down, significant up;

report=;

log/;

proc/;
